# Unmet psychosocial needs in CAPS

**DOI:** 10.1186/1546-0096-13-S1-P175

**Published:** 2015-09-28

**Authors:** G Erbis, T Sergiichuk, S Hansmann, I Haug, SM Benseler, J Kuemmerle-Deschner

**Affiliations:** 1 University Hospital Tuebingen, Department of Pediatrics, Division of Pediatric Rheumatology, Tuebingen, Germany; 2 University of Calgary, Rheumatology, Alberta Children's Hospital, Calgary, Canada

## Introduction

Cryopyrin-associated periodic syndrome (CAPS) is a rare autoinflammatory disease. Generalized manifestations like recurrent fever and fatigue and organ disease like reduced vision, hearing loss, bone deformities and aseptic meningitis impair patients' well-being. While most physical complaints are well defined and managed by effective IL-1 inhibition, areas of psychosocial needs are much less explored and often unsatisfied even in treated patients.

## Objective

To identify unmet needs in the psychosocial support of children and adults with CAPS.

## Patients and methods

A qualitative study of children and adults diagnosed with CAPS cared for at the autoinflammation reference center Tuebingen was performed. Patients and their families were invited to participate in structured focus group interviews grouped according to age and involvement with the disease: children <14 years, adolescents and young adults 14-21 years, adults >21 years; parents; other family members). Open questions were asked to the group. The group discussion was recorded, transcribed to text and analysed for mentioning of certain topics. Frequency of naming and relevance indicated by discussion participants was calculated and graded.

## Results

The five focus groups comprised of 42 individuals including 25 CAPS patients; 10 females, 15 males, including five children, eight adolescents/young adults and 12 adults. In addition unaffected individuals included 14 parents and three other family members. Key domains of unmet needs identified included information about the disease, understanding of patients' needs, intervention in social network and exchange of experiences. The area of need identified in all focus groups and named most often was school. Specifically lack of appreciation by teachers (13) and fellow students (21) was named. In adolescent and adults groups other frequently named areas were employment agency, health insurance organizations and general practitioners.

## Conclusion

Major unmet needs of children and adults with CAPS were identified as various displays of ignorance by the patients' environment. The need for psychosocial support exists particularly in school.

